# Attitudes and knowledge of the tunisian medical staff towards LGBT patients

**DOI:** 10.1192/j.eurpsy.2022.270

**Published:** 2022-09-01

**Authors:** S. Sediri, M. Chahed, I. Bouguerra, A. Maamri, H. Zalila

**Affiliations:** 1 Razi hospital, Emergency And Outpatient Psychiatry Departement, Manouba, Tunisia; 2 Razi Hospital, F Adult Psychiatry Department, Manouba, Tunisia

**Keywords:** stigma, Sexual Minorities, quality of care, medical education

## Abstract

**Introduction:**

Sexual minorities have been coming out more than ever before. However, Tunisian laws and society are still not supportive of LGBT (lesbian, gay, bisexual and transgender) rights. To this day, Tunisian doctors are requisitioned to carry out anal tests as part of expert testimonies in cases of conviction for homosexuality.

**Objectives:**

Assess Tunisian physicians’ attitudes and knowledge towards LGBT patients.

**Methods:**

We conducted a cross-sectional study in October 2021, among 445 Tunisian physicians and medical students. Data were collected via an anonymous self-administered online questionnaire including sociodemographic data and the LGBT Development of Clinical Skills Scale (LGBT-DOCSS).

**Results:**

The overall LGBT-DOCSS score was quite good (4.47 ±0.85). The attitudes of Tunisian doctors were better than their knowledge (p=0.01; t=2.6), which was better than their clinical preparedness (p<10^-3^; t=25) in treating LGBT patients. Doctors who self-identify as sexual minorities and those who interacted with LGBT people in their daily lives, were less stigmatising, more able to treat them and had better knowledge of their needs. Those who had had sexology training (5%) had better LGBT-DOCSS score (p=0.013), better knowledge (p=0.045) and preparedness (p<10^-3^) in treating LGBT patients but did not appear to be less stigmatising than the rest of the group (p=0.9). Religiosity was associated with a more stigmatising attitude (p<10^-3^), but had no impact on knowledge or preparedness.

**Conclusions:**

This study points to gaps, identified by doctors themselves when faced with an LGBT patient. A more inclusive health system requires better matching of health services to the needs of the whole population without discrimination.

**Disclosure:**

No significant relationships.

